# Health-promoting resources and workplace experiences among newly graduated healthcare and social work professionals – a multicentre cross-sectional study

**DOI:** 10.1186/s12913-025-12782-x

**Published:** 2025-04-29

**Authors:** Ingrid Larsson, Inger Ahlstrand, Margaretha Larsson, Sandra Pennbrant, Aimée Ekman, Jenny Hallgren

**Affiliations:** 1 School of Health and Welfare, Halmstad University, PO Box 823, Halmstad, S-301 18 Sweden; 2 School of Health and Welfare, Jönköping University, Jönköping, Sweden; 3 School of Health Sciences, University of Skövde, Skövde, Sweden; 4 Department of Health Sciences, University West, Trollhättan, Sweden

**Keywords:** Health, Health-promoting resources, Healthcare, Social work, Newly graduated, Work experience

## Abstract

**Background:**

Newly graduated healthcare and social work professionals can experience stress and a perceived lack of competence when transitioning from academia to clinical practice, which can lead to health problems or leaving the profession. Globally, creating healthy workplaces remains a challenge. The aim of this study, which had a salutogenic approach, was to explore health-promoting resources and workplace experiences among newly graduated healthcare and social work professionals.

**Methods:**

This multicenter cross-sectional study included Swedish healthcare and social work professionals in their second year after graduation, recruited from six universities. Data were collected in March 2023 via a self-reported, web-based survey using validated instruments: the salutogenically oriented 13-item Sense of Coherence (SOC) Scale, the Salutogenic Health Indicator Scale (SHIS), and the 32-item Work Experience Measurement Scale (WEMS), along with questions on health, well-being, lifestyle and social factors at work, including three from the General Nordic Questionnaire (QPSNordic). Data were analyzed in SPSS 28 with nonparametric tests and Spearman correlations.

**Results:**

A total of 115 newly graduated healthcare and social work professionals completed the questionnaire. The results indicated that those who expressed they were sure to stay in the profession reported a higher total WEMS score (*p* < 0.001), as well as higher scores in five out of six dimensions: supportive working conditions (*p* < 0.001), internal work experience (*p* < 0.001), autonomy (*p* < 0.001), time experience (*p* = 0.006), and management (*p* = 0.029). Participants who rated their well-being as good scored higher in supportive working conditions (*p* = 0.025) and the change process (*p* = 0.008). Those living with children reported higher internal work experience scores (*p* = 0.019).

The results revealed positive and medium-strong to strong correlations between WEMS, SHIS, and SOC total scores. Specifically, there were medium-strong correlations between SHIS and SOC and two dimensions of WEMS: supportive working conditions and time experience, and between SHIS and the change process dimension.

**Conclusions:**

This study highlights the importance of salutogenic resources in supporting newly graduated professionals. Factors such as supportive working conditions, autonomy, and internal work experience were linked to well-being and intention to stay in the profession. Understanding these factors can inform workplace interventions to promote retention and health in early career stages.

## Introduction

It is essential to prioritize the health and well-being of healthcare and social work professionals by addressing challenges, such as workforce shortages, unequal distribution of the workforce, inefficiencies in healthcare delivery, inequities in access to resources, and insufficient support and protection for employees [1]. This current research focuses on healthcare and social work professionals with a bachelor’s degree who work in direct care roles with patients and clients within healthcare and social care settings [2]. Protection and well-being for these professionals are crucial to strengthening a sustainable working life, improving performance, and supporting the delivery of high-quality care [3]. Research shows that healthcare and social work professionals experience job-related stress [4, 5], which can be related to extensive workload and role conflicts, mainly when the professional has limited work experience. In Sweden, ill-health-related stress among this group is increasing [6]. However, positive coping strategies, particularly active coping and help-seeking behavior, are associated with greater well-being and better quality of working life for professionals in these fields [7].

Newly graduated healthcare professionals and less than two years of work experience, in particular, experience stress and face numerous challenges in their roles, including managing various information systems [8] and patient safety [9]. Newly graduated social work professionals face challenges during their first year, such as inadequate workplace orientation, insecurity, unpreparedness for handling distressed clients, uncertainty about their future in the profession constraints that limit client interaction [10]. Budget limitations further restrict the time available for client interaction, contributing to job dissatisfaction [11]. The transition from an academic environment to clinical practice with responsibilities and less support in the work environment among nurses [12] and social workers [10, 13] may contribute to the experience of stress and feelings of being underskilled. This situation can potentially lead to health problems and the decision to leave the profession [12, 14, 15]. To facilitate the development of newly graduated professionals, support from educators, employers in healthcare [16], and social services [10, 13] as well as managers, can promote a positive working climate [13, 17]. Cultivating positive interpersonal relationships is considered vital for new nurses’ career transition and commitment [18]. Similarly, developing stronger workplace induction, along with adequate support and supervision, is seen as helpful in facilitating early-career social workers in their professional roles [10]. In addition to access to supportive work environments and mentorship, personal resilience serves as a protective factor for newly graduated nurses, helping to minimize the negative effects of stressful situations. Strategies such as seeking advice, developing reflective practice, maintaining a positive attitude, and distancing oneself from immediate emotional reactions are recommended to cope with unprofessional behaviors in the workplace [19].

Well-being refers to a state of comfort, health, and happiness [20], and encompasses physical, mental, emotional, and social dimensions [21]. It is influenced by lifestyle choices, environment conditions, and personal relationships [22] and understood as a holistic concept integrating physical, psychological, and social aspects of life [21]. Health is related to human existence and subjective experiences of well-being [23]. It involves a state of balance in life, and relates to balance processes with both an internally and externally focused orientation [24]. In health-promotive research, it is important to apply a holistic description of health. Individual experiences of work and work-life balance are described as essential aspects of health promotion [25]. Healthy lifestyle habits, such as quality sleep, regular physical activity, social relationships, and work-related factors, are positively associated with general health and health-promoting resources [26, 27]. Adequate sleep is essential for optimal health; it is generally recommended that adults aged 18–60 get seven or more hours of sleep per night [28]. However, night shift work can disrupt healthcare professionals’ sleep patterns and quality, thus negatively impacting their psychological health and overall well-being [29]. Research indicates that physical activity levels among healthcare professionals are often insufficient to promote health benefits [30]. Comparatively, nurses tend to participate less in health-promoting activities and have poorer health behaviors than other healthcare professionals [31]. The present study focuses on exploring health‑promoting resources and workplace experiences among newly graduated health and social work professionals.

Health and work-based well-being among professionals in healthcare and social work have been investigated from a range of perspectives. Some studies have explored how insecure employment affects workers’ health and well-being, focusing on factors such as lack of recognition, adverse leadership, high workloads, and limited control over work [32, 33]. Other studies focus on workplace health promotion in health and social care workplaces [34–36]. Workplace health promotion refers to the combined efforts of employers, employees, and health organizations to improve workplace health and well-being and often focuses on promoting healthy lifestyles [35, 37]. Research suggests that individual-focused health promotion measures, such as health profile assessments, lifestyle guidance, and fitness activities, are commonly provided in the workplace [35]. Furthermore, some workplaces adopt a salutogenic approach to health and well-being in healthcare settings [38, 39].

The present study is based on health-promoting resources derived from the salutogenic theory [20]. Health-promoting resources include individual resources and abilities, such as health behaviors and lifestyle factors [40], and aim to promote health rather than focus on cause of disease [41]. Sense of coherence (SOC) is a concept in the salutogenic theory, and includes the dimensions of comprehensibility, manageability, and meaningfulness [42]. Comprehensibility is concerned with having a reflective ability and understanding the whole situation. Manageability is about having the resources to manage different situations, while meaningfulness is the feeling of commitment and motivation for different situations [38, 43]. SOC enables a person to understand the situation and to use the available resources and the generalized and specific resistance resources to stress [20, 44]. In this way, SOC strengthens the ability to assess and understand the situation, find meaning, move in a health-promoting direction, and manage the situation. Previous research shows that the most common reason nurses leave their profession is dissatisfaction (33%), related to understaffing, emotional exhaustion, and impaired patient safety [45].

Research shows that supportive working conditions (i.e., how supported professionals feel at work, including resources, environment, and colleagues), positive time experiences, and internal work experience (i.e., a sense of satisfaction and fulfillment) contribute to good workplace health [46]. Essential health-promoting resources among healthcare professionals encompass positive work experiences, work-life balance, recovery during working hours [25], and supportive relationships with co-workers, managers, and care recipients [47]. It is essential to take a holistic approach to employees throughout their careers to foster a sustainable working life [48]. This study focuses on newly graduated healthcare and social work professionals, exploring how health-promoting resources, healthy lifestyle factors, and workplace experiences contribute to their well-being and long-term career success. Using a salutogenic perspective, it highlights how these resources and experiences foster resilience, promote well-being, and support a sustainable working life, laying a foundation for long-term career fulfillment. Thus, the aim of the present study was to explore health‑promoting resources and workplace experiences among newly graduated healthcare and social work professionals. To address this aim, the following research questions were formulated:

1. What differences in health-promoting resources are associated with sociodemographic characteristics and lifestyle factors?
2. What differences in workplace experiences are associated with sociodemographic characteristics and lifestyle factors?
3. What is the association between health-promoting resources and workplace experiences?

## Method

### Design

This study has a cross-sectional design [49], and forms part of the research project “Health-promoting factors for sustainable working life” [2]. Healthcare and social work professionals who graduated from six universities in Sweden were followed during and after their higher education. The study has been reported according to the Strengthening the Reporting of Observational Studies in Epidemiology (STROBE) [50].

### Sample and setting

A total of 1,751 newly graduated healthcare and social work professionals were invited to participate in the study during their second year after graduation. Of these, 115 completed the questionnaire and were included in the study, resulting in a response rate of 6.6%. The participants were graduates from the following programs: biomedical science, dental hygiene, nursing, occupational therapy, physiotherapy, radiology nursing, and social work. At the time of participation, they had been working in their respective fields for less than two years.

### Data collection

Data collection took place in March 2023, when a self-reported, web-based questionnaire including 136 questions (esMaker NX3 software) was sent to them during the second year of after graduation. The survey was available for approximately eight weeks, during which three reminders were sent. The questionnaire included sociodemographic characteristics (gender, age, ethnicity, and family situation), satisfaction with choice of profession, healthy lifestyle factors, health and health-promoting resources, and workplace experiences.

### Measurements

#### Health‑promoting resources

The Sense of Coherence (SOC) 13-item scale [20] was used to assess health-promoting resources. The SOC-13 scale comprises items related to comprehensibility (five items), manageability (four items), and meaningfulness (four items). SOC-13 uses a seven-point scale, ranging from low (scored as 1) to strong (scored as 7), with a total score ranging from 13 to 91, with a higher score indicating a stronger SOC. SOC-13 demonstrates robust psychometric properties, high validity, and reliability and has proven to be a valuable indicator of health, validated in general adult populations [21].

The Salutogenic Health Indicator Scale (SHIS) 12-item scale adopts a salutogenic perspective on health and assesses cognitive, physical, and psychosomatic health over the preceding four weeks [51]. SHIS uses a six-point scale, ranging from negative (scored as 1) to positive (scored as 6), with a total score ranging from 11 to 66 and higher scores indicating better health. SHIS has demonstrated a high validity, and was validated in a population of healthcare staff [51].

General health and perceived well-being were assessed using two questions: ‘In general, how would you describe your health?’ and ‘In general, how would you describe your well-being?’ Both questions were dichotomized into two categories: excellent/very good/good or less good/bad. Healthy lifestyle factors were assessed using three questions from the Swedish Public Health Survey [52], including physical exercise (dichotomized as yes > 60–90 min/week, no < 30–60 min/week), everyday physical activities, (dichotomized as yes > 150 min/week, no < 150 min/week), sleeping problems (no, yes).

#### Workplace experiences

The Work Experience Measurement Scale (WEMS) 32-item scale has proved to be a workplace health-promotion questionnaire measuring work experiences from a salutogenic perspective [53, 54]. WEMS assesses six dimensions of work experience: supportive work conditions (seven items), internal work experience (six items), autonomy (four items), time experience (three items), management (six items), and change process (six items). WEMS uses a six-point Likert scale ranging from disagree completely (scored as 1) to agree completely (scored as 6) with a total score ranging from 32 to 192. A higher score indicates a positive experience of work. WEMS has demonstrated high validity and reliability and has been validated in a population of healthcare staff [54].

Social factors at work were measured using three questions from the General Nordic Questionnaire (QPSNordic) [55]. The questions assess “co-workers’ listening”, “support from co-workers”, and “good relationships with co-workers”, measured on a five-point Likert scale, ranging from very little (scored as 1) to very much (scored as 5). Sure to stay in the profession were assessed using a single question dichotomized into very sure/quite sure or quite unsure/unsure.

### Data analysis

All data were analyzed using IBM SPSS Statistics 28. Age was categorized into two groups by the median age, ≤ 31 and > 32 years. The variables about which organization they worked in were reported as regional, municipal, or private. All other sociodemographic characteristics and healthy lifestyle variables were dichotomized as Yes and No. The variables “co-workers’ listening”, “support from co-workers”, and “good relationships with co-workers, were dichotomized as yes or no.

The SHIS was calculated and used as one total scale. The SOC and WEMS were calculated and used as total scores, as well as three (SOC) and six (WEMS) subscales. To compare groups, nonparametric tests were used since that data did not fit normal distribution: Mann-Whitney U-test or Chi-square test for two groups and Kruskal-Wallis test for more than two groups. Groups with fewer than 25 participants were not analyzed. To analyze the relationship between WEMS, SHIS, and SOC and included dimensions, Spearman rank-order correlations were calculated. The level of significance was set at 0.05.

## Results

The sample included 115 respondents, 85.2% (*n* = 98) women, and 14.8% (*n* = 17) men, with a total median age of 32 (range 24–58). The respondents worked as registered nurses (*n* = 70), social workers (*n* = 24), occupational therapists (*n* = 9), physiotherapists (*n* = 5), biomedical laboratory scientists (*n* = 3), diagnostic radiology nurses (*n* = 2) and registered dental hygienists (*n* = 2), in regional (48.7%), municipal (31.3%) and private (11.3%) healthcare and social care. Most respondents lived together with someone (81.7%), 30.4% lived with children, and most were born in Sweden (85.2%). The respondents answered that they had, in general, good health (86.1%), and perceived good well-being (80.0%), but the majority had sleeping problems (52.2%). Among respondents, 76.5% reported being sure they would stay in their profession (Table 1).

**Table 1:** Characteristics of participants and those who were sure to stay in the profession

	Total, *n*=115	Sure to stay in the profession, *n* =88 Yes % (*n*)
Age, years, M (SD)	34.1 (8.9)	34.8 (9.1)
Age groups, % (n)		
≤31	48 (55)	43.2 (38)
>32	52 (60)	56.8 (50)
Women, % (n)	85.2 (98)	87.5 (77)
Professions, (n)		
Biomedical laboratory scientist	3	3
Diagnostic radiology nurses	2	2
Registered dental hygienists	2	1
Occupational therapist	9	6
Physiotherapist	5	4
Registered nurse	70	57
Social worker	24	15
Living alone, % (n)	18.3 (21)	15.9 (14)
Children at home, % (n)	30.4 (35)	32.9 (29)
Born in Sweden, % (n)	85.2 (98)	82.9 (73)
General health rated as good to excellent, % (n)	86.1 (99)	86.4 (76)
Well-being rated as good to excellent, % (n)	80.0 (92)	80.7 (71)
Physical exercise (>60 min/week), % (n)	58.3 (67)	55.7 (49)
Everyday physical activities (>150 min/week), % (n)	43.5 (50)	44.3 (39)
No sleeping problems, % (n)	52.2 (60)	48.9 (43)
Satisfied with study choice, % (n)	95.7 (110)	100.0 (88)
Workplace setting, % (n)		
Municipal	31.3 (36)	33.0 (29)
Region	48.7 (56)	51.1 (45)
Private	11.3 (13)	10.2 (9)
Support from colleagues, % (n)	96.5 (111)	96.6 (85)
Colleagues listening, % (n)	94.8 (109)	96.6 (85)
Good relationship with colleagues, % (n)	97.4 (112)	98.9 (87)

### Health‑promoting resources

The total SOC score was 63.77 (SD 12.79). There were no statistically significant differences in total SOC scores, nor in the three dimensions, among the age groups, or between women and men. A higher total SOC score and higher meaningfulness score were found among respondents living with children (*p* = 0.028) and (*p* = 0.007). Respondents who perceived good well-being and did not have sleeping problems had a significantly higher total SOC score (*p* < 0.001) and higher scores in the three dimensions (*p* < 0.001), compared to respondents who reported bad well-being and had sleeping problems. A higher total SOC score (*p* = 0.049) and higher meaningfulness score (*p* = 0.004) were found among respondents who answered that they were sure to stay in the profession (Table 2).

**Table 2:** Total sense of coherence (SOC) and the three dimensions

	SOC Total Mean (SD)	*P*-value	Comprehensibility Mean (SD)	*P*-value	Meaningfulness Mean (SD)	*P*-value	Manageability Mean (SD)	*P*-value
Total (115)	63.77 (12.79)		22.69 (5.58)		21.38 (4.19)		19.71 (4.52)	
Age groups		0.571		0.888		0.318		0.811
≤ 31	63.38 (12.54)		22.64 (5.27)		21.09 (4.20)		19.65 (4.16)	
> 32	64.14 (13.12)		22.73 (5.68)		21.65 (4.19)		19.76 (4.65)	
Gender		*		*		*		*
Women	63.81 (12.35)		22.77 (5.40)		21.42 (4.07)		19.64 (4.33)	
Men	63.53 (15.47)		22.24 (6.70)		21.18 (4.93)		20.12 (5.60)	
Children at home		0.028		0.105		0.007		0.134
Yes	67.59 (11.67)		23.97 (5.26)		22.83(3.73)		20.68 (4.13)	
No	62.13 (12.97)		22.13 (5.66)		20.75 (4.24)		19.29 (4.64)	
Living alone		0.570		0.739		0.292		0.546
Yes	62.24 (13.28)		22.90 (5.44)		20.43 (4.38)		18.90 (5.13)	
No	64.12(12.73)		22.64 (5.64)		21.60 (4.14)		19.89 (4.38)	
Born in Sweden		*		*		*		*
Yes	64.05 (12.56)		22.77 (5.65)		21.38 (4.07)		19.92 (4.40)	
No	61.17 (15.86)		22.00 (6.33)		21.00 (4.60)		18.17 (6.15)	
General health		*		*		*		*
Good	65.56 (11.44)		23.40 (5.31)		21.94 (3.66)		20.22 (4.24)	
Bad	52.07 (15.25)		18.25 (5.27)		17.94 (5.57)		16.33 (4.98)	
Perceived well-being		< 0.001		< 0.001		< 0.001		< 0.001
Good	67.18 (10.64)		23.98 (5.09)		22.36 (3.49)		20.80 (3.88)	
Bad	50.43 (11.89)		17.52 (4.42)		17.48 (4.54)		15.43 (4.39)	
Physical exercise		0.308		0.204		0.573		0.302
Yes	65.14 (11.37)		23.36 (4.91)		21.63 (3.94)		20.17 (4.39)	
No	61.85 (14.46)		21.75(6.33)		21.05 (4.52)		19.06 (4.66)	
Everyday physical activities		0.105		0.114		0.292		0.090
Yes	66.43 (11.21)		23–74 (5.24)		21.94 (3.97)		20.76 (3.92)	
No	61.73 (13.62)		21.88 (5.74)		20.95 (4.32)		18.91 (4.81)	
Sleeping problems		< 0.001		< 0.001		< 0.001		< 0.001
No	68.24 (10.85)		24.28 (5.36)		22.73 (3.62)		21.20 (3.69)	
Yes	58.89 (13.05)		20.95 (5.33)		19.91 (4.30)		18.07 (4.80)	
Satisfaction with study choice		*		*		*		*
Yes	64.22 (12.52)		22.85 (5.46)		21.63 (4.05)		19.74 (4.42)	
No	54.00 (16.17)		19.00 (7.68)		16.00 (3.87)		19.00 (7.04)	
Workplace setting		*		*		*		*
Municipal	65.80 (12.32)		23.61 (5.62)		21.53 (3.81)		20.60 (4.15)	
Region	64.52 (11.44)		22.86 (5.52)		21.91 (3.36)		19.75 (4.19)	
Private	58.83 (17.52)		20.85 (6.05)		19.77 (6.88)		18.67 (6.30)	
Support from colleagues		*		*		*		*
Yes	64.24 (12.41)		22.89 (5.51)		21.48 (4.06)		19.87 (4.35)	
No	51.00 (18.51)		17.00 (5.01)		18.75 (7.27)		15.25 (7.27)	
Colleagues listening		*		*		*		*
Yes	64.50 (11.80)		22.94 (5.34)		21.66 (3.79)		19.90 (4.30)	
No	50.83 (22.41)		18.17 (8.32)		16.33 (7.20)		16.33 (7.20)	
Good relationship with colleagues		*		*		*		*
Yes	64.52 (11.90)		22.96 (5.31)		21.64 (3.88)		19.91 (4.32)	
No	36.33 (17.04)		11.67 (4.04)		11.67 (4.01)		12.33 (6.66)	
Sure to stay in the profession		0.049		0.162		0.004		0.277
Yes	65.31 (11.82)		23.18 (5.40)		22.08 (3.64)		20.02 (4.21)	
No	58.62 (14.72)		21.07 (5.94)		19.11 (5.05)		18.65 (5.39)	

Mann-Whitney Test, Kruskal Wallis Test

*Too small sample size to perform statistical analysis

The total SHIS score was 47.41 (sd 11.49). No statistically significant differences were found among age groups nor between women and men. A significantly higher SIHS score was found among respondents who perceived good well-being compared to respondents who perceived bad well-being (*p* < 0.001). Respondents with no sleeping problems had a higher SHIS total score compared to participants with sleeping problems (*p* < 0.001). Respondents who were sure to stay in the profession had higher SHIS, although it was not significant (*p* = 0.053) (Table 3).

**Table 3:** Total score on salutogenic health Indicator scale (SHIS)

	SHIS Mean (SD)	*P*-value
Total (*n* = 115)	47.41 (11.49)	
Age groups		0.730
≤31	36.36 (9.23)	
>32	36.80 (13.30)	
Gender		*
Women	47.01 (11.20)	
Men	49.71 (13.16)	
Children at home		0.148
Yes	49.43 (11.91)	
No	46.93 (11.26)	
Workplace setting		*
Municipal	49.17 (9.75)	
Region	46.66 (11.74)	
Private	46.85 (14.44)	
Living alone		0.148
Yes	46.24 (10.32)	
No	47.67 (11.77)	
Born in Sweden		*
Yes	47.09 (11.48)	
No	49.24 (11.72)	
General health (Good)		*
Yes	48.93 (10.22)	
No	38.00 (14.51)	
Perceived well-being (Good)		< 0.001
Yes	50.40 (9.41)	
No	35.43 (11.41)	
Physical exercise		0.162
more than 60 min	48.64 (10.58)	
No	45.69 (12.55)	
Everyday physical activities		0.588
more than 150 min	48.38 (10.49)	
No	46.66 (12.23)	
Sleeping problems		< 0.001
No	41.55 (12.01)	
Yes	52.78 (7.83)	
Sure to stay in the profession		0.053
Yes	48.82 (10.42)	
Not sure	42.81 (13.66)	
Satisfaction with study choice		*
Yes	47.95 (10.96)	
No	35.60 (17.46)	
Support from colleagues		*
Yes	47.46 (11.23)	
No	43.25 (19.05)	
Colleagues listening		*
Yes	48.03 (10.66)	
No	37.17 (19.87)	
Good relationship with colleagues		*
Yes	48.04 (10.91)	
No	23.67 (7.23)	

Mann-Whitney Test, Kruskal Wallis Test

*Too small sample size to perform statistical analysis

### Workplace experiences

The total WEMS score was 136.15 (sd 23.19), the dimensions of supportive working conditions 32.33 (sd 6.04), internal work experience 29.40 (sd 5.28), autonomy 14.96 (sd 4.82), time experience 9.55 (sd 4.31), management 25.73 (sd 6.59) and process of change 24.06 (sd 6.86). Respondents living with children had higher internal work experience (*p* = 0.019), compared to respondents who did not live with children. Respondents who perceived good well-being had higher scores in supportive working conditions (*p* = 0.025) and process of change (*p* = 0.008), respectively. Respondents who were sure to stay in the profession had, statistically, a higher total score in WEMS (*p* < 0.001), the dimensions of supportive working conditions (*p* < 0.001), internal work experience (*p* < 0.001), autonomy (*p* < 0.001), time experience (*p* = 0.006) and management (*p* = 0.029), compared to respondents not sure to stay in the profession (Table 4).

**Table 4:** Total work experience measurement scale (WEMS) and the six dimensions

	WEMS Total		Supportive working conditions		Internal work experience		Autonomy		Time experience		Management		Process of change	
	Mean (SD)	*P* value	Mean (SD)	*P* value	Mean (SD)	*P* value	Mean (SD)	*P* value	Mean (SD)	*P* value	Mean (SD)	*P* value	Mean (SD)	*P* value
Total (*n* = 115)	136.15 (23.19)		32.33 (6.04)		29.40 (5.28)		14.96 (4.82)		9.55 (4.31)		25.73 (6.59)		24.06 (6.86)	
Age groups		0.376		0.800		0.381		0.436		0.682		0.595		0.883
≤31	135.16 (22.45)		32.35 (5.62)		29.00 (5.30)		14.62 (4.73)		9.69 (4.16)		25.51 (6.47)		24.00 (7.52)	
>32	137.11 (24.06)		32.32 (6.46)		29.78 (5.29)		15.27 (4.93)		9.42 (4.47)		25.93 (6.75)		24.12 (6.24)	
Gender		*		.*		*		*		*		*		*
Women	135.89 (22.48)		32.13 (6.00)		29.57 (4.87)		15.04 (4.71)		9.40 (4.18)		25.53 (6.83)		23.79 (6.81)	
Men	137.75 (27.91)		33.47 (6.31)		28.38 (7.47)		14.47 (5.57)		11.38 (5.08)		26.94 (4.92)		25.69 (7.15)	
Children at home		0.099		0.053		**0.019**		0.174		0.140		0.719		0.088
Yes	141.24 (19.93)		34.09 (4.70)	.	31.15 (4.00)		15.86 (4.52)		10.46 (4.54)	.	26.00 (6.38)		24.29 (5.71)	
No	134.03 (24.24)		31.59 (6.41)		28.66 (5.60)		14.56 (4.92)		9.15 (4.17)		25.61 (6.72)		23.96 (7.33)	
Living alone		*		*		*		*		*		*		*
Yes	134.24 (18.27)		31.67 (5.36)		29.76 (4.52)		14.19 (4.20)		8.38 (3.81)		26.10 (6.60)		24.14 (7.30)	
No	136.59 (24.26)		32.48 (6.20)		29.32 (4.52)		15.13 (4.96)		9.81 (4.38)		25.65 (6.62)		24.04 (6.80)	
Born in Sweden		*		*		*		*		*		*		*
Yes	135.77 (23.45)		32.48 (5.94)		29.49 (5.47)		24.73 (4.00)		9.49 (4.31)		25.49 (6.70)		23.67 (7.08)	
No	138.44 (22.18)		31.47 (6.72)		28.88 (4.08)		16.24 (5.51)		9.88 (4.39)		27.19 (5.88)		26.44 (4.84)	
General health (Good)		*		*		*		*		*		*		*
Yes	137.15 (21.10)		32.76 (5.78)		29.63 (4.65)		14.92 (4.72)		9.52 (4.22)		25.77 (6.56)		24.41 (6.62)	
No	129.67 (34.08)		29.53 (7.14)		28.00 (8.25)		15.19 (5.61)		9.75 (4.97)		25.50 (6.97)		21.94 (8.09)	
Perceived well-being (Good)		0.081		**0.025**		0.092		0.941		0.148		0.456		**0.008**
Yes	139.03 (19.51)		33.10 (5.54)		30.02 (4.46)		14.91 (4.95)		9.82 (4.16)		26.07 (6.24)		24.93 (6.43)	
No	124.36 (32.40)		29.14 (7.09)		29.96 (7.38)		15.13 (4.36)		8.48 (4.81)		24.39 (7.85)		20.65 (7.55)	
Physical exercise		0.193		0.287		0.924		**0.016**		0.163		0.464		0.065
Yes	134.45 (21.58)		31.96 (5.81)		29.53 (4.81)		14.04 (5.17)		9.09 (4.32)		25.41 (6.43)		24.86 (7.03)	
No	138.59 (25.39)		32.87 (6.38)		29.23 (5.93)		16.23 (4.01)		10.19 (4.25)		26.17 (6.86)		22.94 (6.53)	
Everyday physical activities		0.292		0.361		0.846		0.852		0.369		0.241		0.206
Yes	138.24 (23.58)		32.72 (6.37)		29.62 (5.00)		14.78 (4.71)		9.96 (4.20)		26.46 (6.75)		24.70 (7.50)	
No	134.47 (22.94)		32.03 (5.80)		29.23 (5.53)		15.09 (4.40)		9.23 (4.39)		25.16 (6.47)		23.56 (6.33)	
Sleeping problems		0.399		0.619		0.078		0.805		0.091		0.993		0.194
No,	139.08 (19.75)		32.88 (5.58)		30.32 (4.48)		14.95 (4.89)		10.18(4.02)		25.83 (6.33)		24.92 (6.38)	
Yes	132.77 (26.42)		31.72 (6.51)		28.39 (5.93)		14.96 (4.80)		8.85 (4.54)		25.61 (6.93)		23.09 (7.31)	
Satisfaction with study choice		**0.013**		**0.019**		**0.021**		**0.009**		**0.039**		0.197		0.580
Yes	137.55 (22.04)		32.63 (5.88)		29.75 (4.81)		15.25 (4.64)		9.72 (4.30)		25.88 (6.63)		24.17 (6.69)	
No	106.20 (29.66)		25.80 (6.97)		21.80 (9.36)		8.60 (4.78)		5.80 (2.78)		22.40 (5.18)		21.80 (10.73)	
Workplace setting		*		*		*		*		*		*		*
Municipal	137.67 (17.99)		31.97 (5.39)		29.51 (4.36)		16.67 (5.12)		10.31(4.59)		25.46 (5.89)		23.18 (6.54)	
Region	139.39 (22.82)		33.43 (5.39)		30,57 (4.64)		14.18 (4.47)		9.30 (4.15)		26.43 (6.41)		25.48 (6.49)	
Private	121.15 (30.19)		28.62 (9.01)		25.85 (8.08)		14.08 (4.79)		9.23(4.75)		21.38 (7.95)		22.00 (7.16)	
Support from colleagues		*		*		*		*		*		*		*
Yes	137.25 (22.27)		32.61 (5.65)		29.49 (5.19)		15.06 (4.65)		9.64 (4.18)		25.82 (6.63)		24.25 (6.71)	
No	96.33 (25.48)		24.75(11.70)		26.33 (8.96)		12.00 (8.83)		7.00 (7.35)		22.33 (4.73)		17.33(10.60)	
Colleagues listening		*		*		*		*		*		*		*
Yes	137.79 (21.33)		32.67 (5.81)		29.82 (4.64)		15.00 (4.69)		9.70 (4.29)		25.99 (6.60)		24.46 (6.55)	
No	101.20 (35.79)		25.00 (6.96)		21.83 (9.87)		14.17 (7.41)		6.83 (4.07)		21.00 (4.56)		17.00 (8.99)	
Good relationship with colleagues		*		*		*		*		*		*		*
Yes	138.07 (20.34)		32.74 (5.58)		29.86 (4.52)		15.13 (4.77)		9.66 (4.29)		26.01 (6.45)		24.53 (6.33)	
No	66.33 (4.40)		17.33 (0.58)		12.67 (4.93)		8.67 (2.08)		5.33 (2.52)		15.33 (2.08)		7.00 (1.73)	
Sure to stay in the profession		**<** ** 0.001**		**< 0.001**		**< 0.001**		**< 0.001**		**0.006**		**0.029**		0.060
Yes	141.68 (20.43)		33.53 (5.37)		30.46 (4.56)		15.92 (4.39)		10.16(4.28)		26.45 (6.53)		24.87 (6.26)	
No	118.74 (23.10)		28.48 (6.55)		26.00 (6.08)		11.81 (4.91)		7.56 (3.82)		23.41 (6.37)		21.48 (8.09)	

Mann-Whitney Test, Kruskal Wallis Test

*Too small sample size to perform statistical analysis

### Associations between health‑promoting resources and workplace experiences

For this sample, all three scales had good internal consistency with Cronbach’s alpha coefficient of 0.93 (WEMS), 0.93 (SHIS) and 0.89 (SOC). Correlations were performed between the total scores of respectively WEMS, SHIS and SOC and the associated dimensions of WEMS and the associated dimensions of SOC. Correlations between the total score of WEMS, SHIS, and SOC were positive and medium-strong to strong. Correlations between SHIS and the dimensions of WEMS, the process of change, supportive work conditions, and time experience were positive and medium-strong. Correlations between SHIS and SOC total and the three respective salutogenic dimensions were positive and strong. Correlations between SOC total and the dimension of WEMS in supportive work conditions and time experience were positive and medium-strong (Table 5).

**Table 5:** Associations between WEMS, SHIS, and SOC and included dimensions

	WEMS								SOC		
	WEMS Total	Process of change	Management	Internal Work experiences	Supportive Work conditions	Autonomy	Time experience	SHIS	SOC Total	SOC Comprehensibility	SOC Meaningfulness
Process of change	.726^**^										
Management	.759^**^	.547^**^									
Internal work experiences	.539^**^	.299^**^	.253^**^								
Supportive work conditions	.785^**^	.490^**^	.538^**^	.497^**^							
Autonomy	.462^**^	.117	.244^**^	.313^**^	.423^**^						
Time experience	.416^**^	.193	.125	.089	.245^*^	.370^**^					
SHIS Total	.379^**^	.304^**^	.172	.241^**^	.395^**^	.322^**^	.413^**^				
SOC Total	.267^*^	.289^**^	.091	.215^*^	.322^**^	.086	.308^**^	.623^**^			
Comprehensibility	.217^*^	.244^**^	.097	.104	.255^**^	.012	.215^*^	.580^**^	.910^**^		
Meaningfulness	.256^*^	.261^**^	.103	.321^**^	.313^**^	.075	.197	.490^**^	.811^**^	.590^**^	
Manageability	.257^*^	.295^**^	.057	.188^*^	.288^**^	.095	.343^**^	.553^**^	.890^**^	.743^**^	.612^**^

**Correlation is significant at the 0.01 level, *Correlation is significant at the 0.05 level

## Discussion

In this study, a salutogenic perspective was applied to explore health-promoting resources and workplace experiences among newly graduated healthcare and social work professionals. The findings highlight statistically significant correlations between health-promoting resources and experienced work environments, suggesting that a focus on these resources can enhance health and support a sustainable working life. Additionally, the results demonstrate significant positive associations between the SOC, SHIS, and WEMS scores, indicating that these instruments can together provide a more comprehensive understanding of work-related health.

### Health‑promoting resources

The results demonstrated positive and strong correlations between SHIS and SOC total scores and the three dimensions of comprehensibility, manageability, and meaningfulness among newly graduated healthcare and social work professionals.

Those who lived with children and were sure to stay in the profession reported higher total SOC and meaningfulness scores. This result may suggest that enthusiasm for and commitment to the work remain intact, the work situation is manageable, and a healthy balance between work and family life promotes resilience. Research has shown that family size, specifically the number of children at home, is not significantly associated with work-family conflict or the desire to remain in the profession. Balancing work demands and resources is crucial for managing workplace stress and enhancing work engagement [17]. A higher SHIS score was also found among those who were sure to stay in the profession. This result may indicate that newly graduated healthcare and social work professionals find the profession interesting and meaningful. This includes having a sense of connection and community with others and influence and control over one’s work situation [56]. Additionally, their desire to remain in the profession could be linked to the feeling that their work contributes to their overall life purpose [57].

Those who perceived good well-being and those without sleeping problems reported higher total SHIS and SOC scores (*p* < 0.001), along with higher scores in comprehensibility, manageability, and meaningfulness. Previous research indicates that sleep is a crucial health-promoting factor for newly graduated registered nurses, particularly during their transition from student to professional practitioner [58, 59]. Research shows that a clinical introduction program can support newly graduated nurses in their transition into the profession, by incorporating reflection and guiding them through phases of uncertainty, security, challenge, and growth as they develop their new professional identity [60].

### Workplace experience

By focusing on factors that promote workplace health and well-being from a salutogenic perspective, the WEMS provides a comprehensive assessment of work experiences [53, 54]. This study identified that several workplace health-promotion resources are crucial in relation to the sociodemographic characteristics and health-promoting resources of newly graduated healthcare and social work professionals. The results can be compared with earlier studies that reported the total WEMS score and subscales for almost the same target group [46, 53, 61, 62]. Those who were sure to stay in the profession reported more positive work experience, as evidenced by the total WEMS score (*p* < 0.001). The results also revealed positive and medium-strong to strong correlations between WEMS, SHIS, and SOC total scores.

The dimension of supportive work conditions assesses how supported professionals feel in their workplace, encompassing factors such as resource availability, a supportive environment, and the level of colleague assistance and encouragement. It evaluates the workplace experience, enabling professionals to carry out their duties effectively and comfortably [53, 54]. Newly graduated healthcare and social professionals who reported higher supportive work conditions perceived good well-being and were sure to stay in the profession. The results show medium-strong correlations between higher supportive work conditions and higher SHIS and SOC. Newly graduated nurses highlight the importance of supportive work conditions, including advice, patience, and selflessness from colleagues, supervisors, and managers [63–65]. They also highly value collaboration with experienced colleagues who offer guidance and support, ensuring that they have someone to turn to for assistance or clarification, which fosters a sense of safety and security in their work environment [66, 67]. Previous research highlights the crucial requirement for strong support systems for newly graduated professionals. It advocates assigning senior nurses with clinical teaching qualifications and sufficient educational resources to mentor new nurses effectively, offering essential guidance and support during their transition into clinical practice [67–69]. According to Theorell and Karasek (1996), workload, level of decision-making power, and ability to apply one’s skills are environmental factors that can lead to varying levels of work stress. From a health-promotion perspective, these factors are key contributors to workplace well-being and job satisfaction, as they directly affect an individual’s sense of control, autonomy, and fulfillment in their professional role. Effective health-promotion involves contextualizing these issues to make them more understandable and meaningful. Consequently, active participation becomes crucial in successful health-promoting initiatives [70]. A supportive work environment means that healthcare managers provide diverse clinical experiences through job rotation, fulfill promises made during induction, and foster a culture of trust. This enriched environment and consistent support are crucial for new graduate nurses and can significantly improve professional retention [66]. These factors are essential for navigating challenging situations, and problem-focused coping strategies improve with professional experience [63]. Research indicates that newly graduated nurses are highly committed to continuous learning in the workplace, often relying on colleague support to enhance this process. To support their ongoing professional development, the implementation of well-designed and structured interventions is recommended [64, 71, 72].

The dimension of time experience assesses professionals’ perceptions of time utilization at work, considering factors such as time pressure, workload balance, and meeting deadlines. A positive time experience suggests effective time management and a well-balanced workload [53, 54]. Newly graduated healthcare and social work professionals who were sure to stay in the profession reported higher scores in the dimension of time experience. The results also show medium-strong correlations between SHIS, SOC, and the time experience dimension. However, this study found time experience to be lower than in previous research [46, 53, 61, 62]. This discrepancy may be explained by the relative lack of experience among newly graduated healthcare and social work professionals, which could lead to difficulties in managing their tasks effectively. Research indicates that newly graduated registered nurses often find the transition to clinical practice overwhelming, especially when it comes to effectively managing their time [67].

The dimension of the change process assesses how professionals experience and manage organizational changes, including the transparency of the process, involvement in decision making, and the support received during transitions. A positive score on the change process indicates that professionals feel well-informed, engaged in decision making, and supported throughout organizational transitions [53, 54]. Those in our current study who perceived good well-being reported higher scores in the dimension of the change process. The results indicate medium-strong correlations between SHIS and the change process dimension. Previous research has established that organizational changes are associated with reduced employee well-being [73]. For a change to be considered successful, it is important that healthcare professionals have the opportunity to influence the change, are adequately prepared for it and recognize its value [74]. Additionally, research highlights the importance of managing employees’ perceptions of change [75].

The dimension of internal work experience focuses on the underlying aspects of work that contribute to professionals’ sense of satisfaction and fulfillment. This includes feelings of accomplishment, recognition, personal growth, and the meaningfulness of the work performed. Internal work experience captures how the work enhances professionals’ well-being and motivation [53, 54]. Higher levels of internal work experience were reported among those who were sure to stay in the profession and those living with children. Internal work experience is linked to the meaningfulness of professional roles, and newly graduated registered nurses perceive nursing as fulfilling and meaningful [67]. This aligns with previous research, which indicates that high levels of perceived empowerment among newly graduated nurses is associated with high satisfaction with the employing institution and the opportunity to develop career plans [63]. To the best of our knowledge, there is little to no existing research that might help explain the finding in our current study – that individuals living with children reported higher levels of internal work experience. Further research is needed to explore this relationship in more depth.

The dimension of autonomy assesses the degree of independence and control professionals have over their work. This includes their ability to make decisions, organize and prioritize tasks, and influence their work environment. High autonomy is often linked with increased job satisfaction and empowerment [53, 54]. This study demonstrates that those who were sure to stay in the profession reported higher levels of autonomy. Research indicates that most newly graduated nurses experience high job satisfaction, which is linked to both intrinsic factors, such as autonomy and the meaningfulness of their work, and extrinsic factors. Structural empowerment and career satisfaction significantly enhance job satisfaction, which tends to increase over time and contribute to the overall well-being of newly graduated nurses [12]. Healthcare and social work professionals are often engaged in interdisciplinary collaboration, which requires autonomy within their respective fields. This collaboration involves integrated decision-making, which is developed and refined through the professionalization process [76]. Newly graduated nurses express a strong sense of responsibility and professional autonomy at this early stage of their careers [77]. Previous research indicates that newly graduated nurses perceive their levels of empowerment and competence as moderate [63] to high [78], correlating positively with job satisfaction, and were sure to stay in their current positions [78]. Empowerment perceptions are influenced by various factors, including individual characteristics, organizational dynamics, and the work environment [63].

The dimension of management assesses the quality of leadership at the workplace, encompassing communication, decision-making, support from managers, and clarity regarding organizational goals and expectations. Competent leadership is essential in promoting a positive work experience [53, 54]. In this study, those participants who were sure to stay in the profession scored highly in the dimension of management. In a national survey of occupational therapists, insufficient leadership and limited support from managers were described as contributing to the consideration of leaving the profession [79].

The findings of this study have several important implications for practice. First, it is essential that healthcare and social work organizations invest in resources that promote health, such as adequate staffing, training, and support systems. Developing supportive work conditions, including colleague assistance, supervisor support, and structured mentorship, should be prioritized to help newly graduated professionals navigate the transition into their roles. Additionally, improving work-life balance, particularly by considering family dynamics, can enhance professionals’ sense of meaningfulness and well-being, thus promoting retention in the profession. To foster a sustainable workforce, organizations should also focus on enhancing autonomy, decision-making opportunities, and leadership support, as these are critical to job satisfaction and career longevity. Finally, implementing structured programs that address the change process in organizations and offer clear communication and support can significantly improve professionals’ well-being and engagement in their roles.

### Strengths and limitations

The study’s strength lies in the multicenter cross-sectional design, the use of various validated instruments, and the inclusion of newly graduated professionals from diverse healthcare and social work programs at six universities in western Sweden. Another strength is that the study is part of a larger research project, whose study protocol has been reviewed and published, to enhance research standards and reduce publication bias [2]. The validated instruments used in the current study were specifically developed to capture health-promoting resources. Additionally, there is limited research focusing on healthcare and social care that predict salutogenic factors rather than disease-causing factors.

The study’s limitations include a relatively low response rate, despite a total sample of all eligible graduating healthcare and social work professionals from six universities being invited. The resulting small sample size, with low male participation limits the possibility of comparisons across different professions and genders.

## Conclusion

This study indicates that newly graduated healthcare and social work professionals generally experience good health and well-being. Higher scores on SOC, SHIS, and WEMS were associated with access to health-promoting resources, a supportive work environment, and favorable work-related health. When used together, these instruments may contribute to a broader understanding of how work-related stress is perceived and managed.

These findings suggest that resource availability, a supportive environment, and peer and supervisor support may be relevant for the well-being of newly graduated professionals. Although further research is needed to explore how these aspects relate to long-term career sustainability, job satisfaction, and quality of care, the results may have clinical implications. Supporting newly graduated professionals in their transition to working life may be important for their long-term engagement and ability to provide high-quality care. Initiatives aimed at strengthening workplace support, ensuring access to necessary resources, and promoting professional well-being could be considered in efforts to create sustainable work environments. Such approaches may also contribute to professional resilience and staff retention within healthcare and social care.

## Data Availability

The data is available upon reasonable request. For access to the underlying research data, please contact the corresponding author.
